# Breast cancer characteristics and surgery among women with Li‐Fraumeni syndrome in Germany—A retrospective cohort study

**DOI:** 10.1002/cam4.4300

**Published:** 2021-09-26

**Authors:** Nathalie Rippinger, Christine Fischer, Hans‐Peter Sinn, Nicola Dikow, Christian Sutter, Kerstin Rhiem, Sabine Grill, Friedrich W. Cremer, Huu P. Nguyen, Nina Ditsch, Karin Kast, Simone Hettmer, Christian P. Kratz, Sarah Schott

**Affiliations:** ^1^ Department of Gynecology and Obstetrics University Hospital Heidelberg Heidelberg Germany; ^2^ Institute of Human Genetics University Hospital Heidelberg Heidelberg Germany; ^3^ Department of Pathology University Hospital Heidelberg Heidelberg Germany; ^4^ Center for Hereditary Breast and Ovarian Cancer Center for Integrated Oncology (CIO) Medical Faculty University Hospital Cologne Cologne Germany; ^5^ Department of Gynecology and Centre for Hereditary Breast and Ovarian Cancer Comprehensive Cancer Center (CCC TUM) University Hospital Rechts der Isar Technical University of Munich (TUM) Munich Germany; ^6^ SYNLAB Center for Human Genetics Mannheim Germany; ^7^ Institute of Medical Genetics and Applied Genomics University Hospital of Tuebingen Tuebingen Germany; ^8^ Department of Human Genetics University of Bochum Bochum Germany; ^9^ Department of Gynecology and Obstetrics Ludwig‐Maximilians University (LMU) University Hospital of Munich Munich Germany; ^10^ Department of Gynecology and Obstretrics University Hospital Augsburg Augsburg Germany; ^11^ Department of Gynecology and Obstetrics Medical Faculty University Hospital Carl Gustav Carus Technical University Dresden Dresden Germany; ^12^ National Center for Tumour Diseases (NCT) Partner Site Dresden Dresden Germany; ^13^ Department of Paediatrics and Adolescent Medicine Division of Paediatric Haematology and Oncology Medical Center Faculty of Medicine University of Freiburg Freiburg Germany; ^14^ Paediatric Haematology and Oncology and Rare Disease Program Hannover Medical School Hannover Germany

**Keywords:** breast surgery, cancer predisposition, hereditary breast cancer, Li‐Fraumeni syndrome, mastectomy, prophylactic surgery, *TP53*

## Abstract

**Background:**

Women with Li‐Fraumeni syndrome (LFS) have elevated breast cancer (BC) risk. Optimal BC treatment strategies in this population are yet unknown.

**Methods:**

BC subtypes and treatment were retrospectively investigated between December 2016 and January 2019 in a multicentre study. BC risks were evaluated according to the type of surgery.

**Results:**

Thirty‐five women of our study population (35/44; 79.5%) had developed 36 breast lesions at first diagnosis at a mean age of 34 years. Those breast lesions comprised 32 invasive BCs (89%), three ductal carcinoma in situ alone (8%) and one malignant phyllodes tumour (3%). BCs were mainly high‐grade (18/32), of no special type (NST; 31/32), HER2‐enriched (11/32) or luminal‐B‐(like)‐type (10/32). Affected women (*n* = 35) received breast‐conserving surgery (BCS, *n* = 17) or a mastectomy (ME, *n* = 18) including seven women with simultaneous contralateral prophylactic mastectomy (CPM) at first diagnosis. Nineteen women suffered 20 breast or locoregional axillary lesions at second diagnosis with mean age of 36. Median time between first and second diagnosis was 57 months; median time to contra‐ and ipsilateral recurrence depended on surgical strategies (BCS: 46 vs. unilateral ME: 93 vs. bilateral ME > 140 months). Women with a primary treatment of solitaire therapeutic ME suffered from contralateral BC earlier compared to those with therapeutic ME and CPM (median: 93 vs. >140 months).

**Conclusion:**

Aggressive BC subtypes occur among women with LFS. Surgical treatment, i.e. ME and CPM, may prolong time to a second BC diagnosis. Conclusion on long‐term survival benefit is pending. Individual competing tumour risks and long‐term outcomes need to be taken into consideration.

## Graphical abstract

Women with Li‐Fraumeni Syndrome face challenging breast cancer risks. Therapeutic or contralateral prophylactic mastectomy can be associated with a prolonged median time to develop contralateral breast cancer.

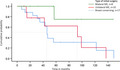

## 1 INTRODUCTION

Li‐Fraumeni syndrome (LFS, MIM 151623) is a rare and highly penetrant cancer predisposition syndrome characterised by a broad cancer spectrum and caused by pathogenic/likely pathogenic (P/LP) germline variants in the *TP53* tumour suppressor gene.^1^, ^2^, ^3^, ^4^ Since *TP53* is included on gene panels offered to women with suspected hereditary breast and ovarian cancer (HBOC), P/LP *TP53* variant carriers are increasingly identified among women not meeting current LFS testing criteria.^1^, ^2^, ^3^, ^5^ Given the fact that not all patients with LFS are being diagnosed the precise number is unknown. Based on published population prevalence estimates of *TP53* germline variants, we estimate that the number within Germany is around 15000.^6^ P/LP in *TP53* are detected in up to 7.7% of women with breast cancer (BC) younger than 31 and in <1% of BC patients independent of family history or age.^7^, ^8^, ^9^, ^10^, ^11^, ^12^, ^13^, ^14^ A recent analysis showed a low prevalence of 0.05% for *TP53* germline variants in BC patients and its association with BC.^15^ Women with LFS face a lifetime BC risk of >50% by age of 70, typically diagnosed in women under the age of 31 and often occurs as synchronous bilateral or metachronous contralateral BC and comes with a general cancer risk of up to 100%.^2^, ^5^, ^10^, ^11^, ^15^, ^16^, ^17^, ^18^, ^19^ In case of a BC diagnosis under the age of 35, annual rates of contralateral BC are almost twice as high among women with LFS compared to female carriers of a P/LP variant in *BRCA1* or *BRCA2* (7.0% vs. 3.6% or 2.6%).^17^ Based on these BC risks and considering the risk for other LFS‐associated cancers such as sarcomas, adrenocortical carcinoma, brain tumours or leukaemia, international experts recommend a comprehensive cancer surveillance programme including annual whole body and breast magnetic resonance imaging (MRI).^20^, ^21^ In cancer‐free women with LFS, bilateral prophylactic mastectomy (BPM) can be considered. In a case of BC diagnosis in a patient with LFS, the knowledge about a P/LP *TP53* germline variant influences the therapeutic approach as radiation and its risk to induce secondary malignancies should be discussed.^22^, ^23^, ^24^, ^25^ Mastectomy (ME) and contralateral prophylactic mastectomy (CPM) might be options to prevent local BC recurrence or contralateral BC.^17^, ^22^, ^26^, ^27^ There is little evidence about surgical approaches, prophylactic operations (PO) and LFS‐specific BC characteristics besides the described high frequency of HER2‐amplified BC subtypes.^15^, ^25^, ^28^, ^29^, ^30^, ^31^, ^32^ To date, LFS is not addressed concretely in the German BC S3 guideline. Recent clinical approaches are based on international recommendations and best experts’ opinions. Here, we retrospectively reviewed pathological and clinical aspects of primary LFS‐associated BCs and of a second BC diagnosis. We analysed breast surgery in respect to surgical techniques and related local recurrence rates in women with LFS in Germany.

## 2 METHODS

### 2.1 Study population and protocol

Women with a P/LP *TP53* variant were identified and contacted between December 2016 and January 2019 by cooperating centres. A subset of the cohort had participated in our previous study.^33^ As previously described, the number of contacted individuals were reported to the initiating centre in Heidelberg^33^: In brief, study information including a study‐specific questionnaire with a pseudonymised code was sent to eligible women. Study participants were included in the current retrospective analysis if complete clinical as well as pathology reports and informed written consents were available as well as filled questionnaires on prophylactic surgery. The study was approved by the institutional review board (S‐370/2016).

Pathological tumour stages were evaluated according to the American Joint Committee on Cancer Anatomic Stage Group. In case of BC recurrence with no axillary procedure, lymph node status was considered negative.^34^ The detection of micrometastasis in lymph nodes was classified as nodal positive. Evaluation of hormone receptor (ER, PgR) and HER2 status, and scoring of Ki‐67 was performed as routine diagnostic by local pathology institutes. Institutions were adherent to pertinent international guidelines at the time of diagnosis, especially the WHO Classification of Tumours framework.^35^

The surrogate molecular subtype was determined according to routine IHC results with Ki67 <20% as the threshold for the distinction between Luminal‐A and Luminal‐B subtypes according to the St. Gallen guidelines 2013.^36^

### 2.2 Satisfaction with prophylactic operations

The applied PO and its respective satisfaction i.e. any type of prophylactic mastectomy (PM, including BPM + CPM) and prophylactic bilateral salpingo‐oophorectomy (PBSO) were evaluated with six self‐designed questions. The response categories ranged on a five‐point scale from “absolutely satisfied” to “totally unsatisfied”. In case of BC, we considered not only CPM in case of first BC but also secondary PM according to the patient´s choice due to the genetic test result or a second BC diagnosis.

### 2.3 Statistical analyses

Statistical analyses and p‐values and were considered to be mainly descriptive. We performed two‐sided *t*‐tests and Fisher‐Yates tests to identify differences between groups. Survival analyses were assessed by estimation of Kaplan–Meier distributions and comparison of subgroups by log‐rank tests. Using Kaplan–Meier curves, the time from first breast lesion to one of the following events: (1) ipsilateral BC and/or DCIS, (2) contralateral BC and/or DCIS, (3) both or (4) the end of observation time was evaluated and distributions in respect to the surgical techniques were compared. In a second step, only contralateral BC and/or DCIS events in formerly ME patients were considered in order to evaluate the potential benefits of a simultaneous CPM in case of BC and/or DCIS. If the month or day was excluded from the date in the medical records, we used January or the first of the month respectively for statistical calculations. One participant with simultaneous bilateral BC was excluded from these subanalyses. *p*‐values <0.05 were considered statistically significant. We used Microsoft Excel Version 15.31 (170216) and SPSS 24 (SPSS Statistics V24, IBM Corporation).

## 3 RESULTS

### 3.1 Study population, breast lesions at first diagnosis and their respective histopathology

**FIGURE 1:**
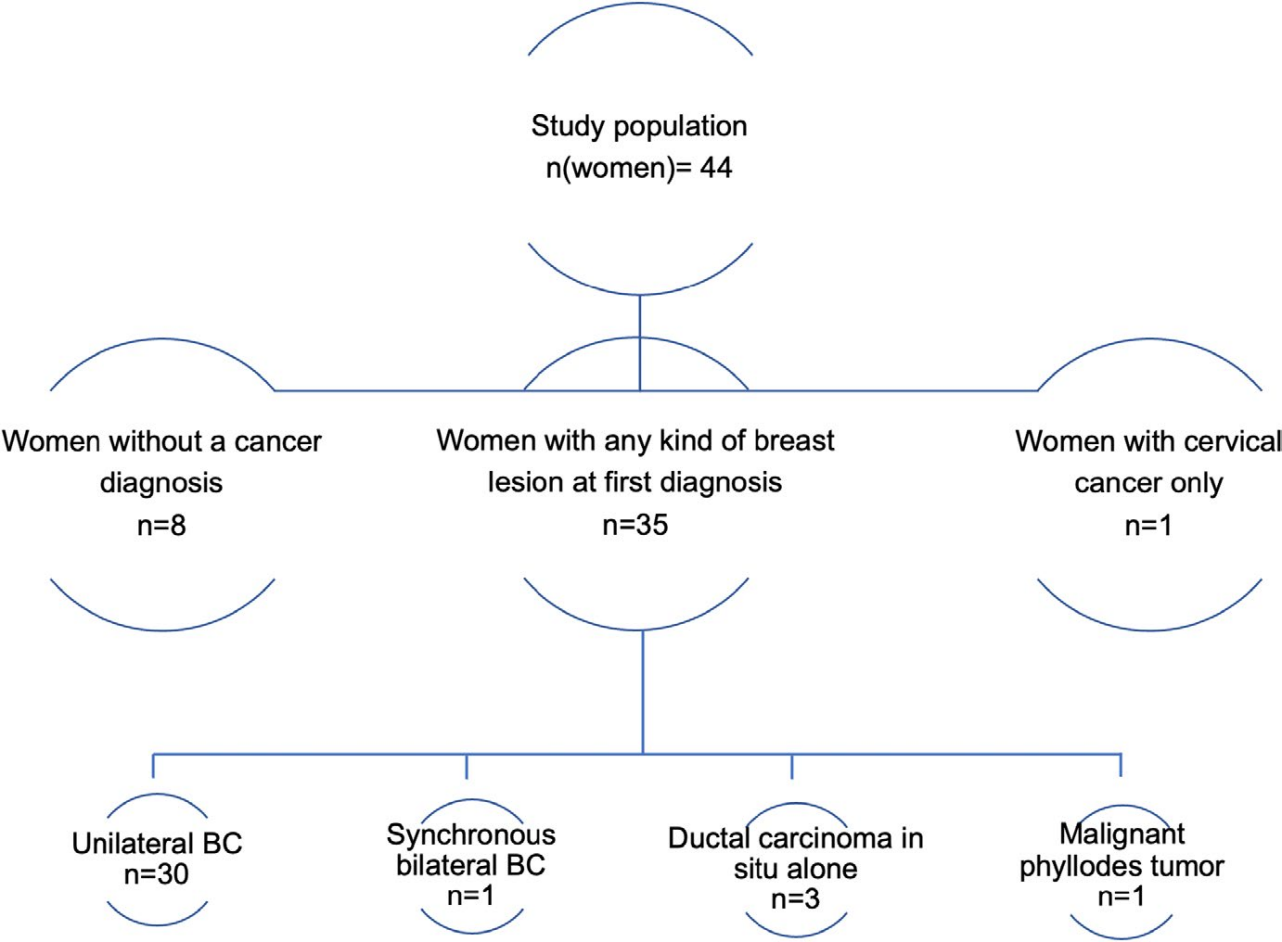
Flowchart of the study population distinguishing affected women and respective breast lesions at first diagnosis

The study population consisted of 44 women with LFS. Socio‐demographic data and personal cancer history are shown in Table 1. Of the 44 women, eight women (18%) were healthy and one (2%) had a solitary cervical carcinoma. Thirty‐five women of the study population (35/44; 80%) had 36 breast lesions (including invasive BC and non‐invasive breast lesions) at a mean age of 34 years old (range: 22–53 years). Thirty of these 35 women (86%) had primary unilateral invasive BC and one (3%) had synchronous bilateral BC leading to a total number of 32 invasive BC lesions in 31 women. The remaining four women had singular ductal carcinoma in situ (DCIS: *n* = 3, 9%) or a malignant phyllodes tumour (*n* = 1, 3%; Figure 1).

**TABLE 1:** Clinical characteristics and cancer history of 44 women with LFS

Study population, *n* = 44	*n* (%)
Age, years (mean, SD, median)	39.3 ± 10.9, 40.0
Country of origin	
Germany	37 (84)
Other	7 (16)
Personal cancer history	
None	8 (18)
Single cervical cancer	1 (2)
Single BC^a^	12 (27)
Multiple BC^b^	14 (32)
Multiple BC + metachronous distant metastasis	2 (6)
Other cancers^c^ + BC	6 (13)
Other cancers^c^ + BC + metachronous distant metastasis	1 (2)

Abbreviations: BC, breast cancer; SD, standard deviation.

^a^ Including singular DCIS and malignant phyllodes tumour.

^b^ Including synchronous or metachronous bilateral BC, BC/DCIS recurrence, and contralateral DCIS/BC.

^c^ Including other primary cancers from the LFS spectrum.

At first diagnosis, invasive BCs (*n* = 32, 91%) were mainly invasive carcinoma of no special type (NST: *n* = 31/32, 97%; with NST + DCIS: *n* = 10, 32% vs. NST alone: *n* = 21, 68%) and one medullary carcinoma (*n* = 1, 3%). Tumours were mainly high‐grade ductal (Grade 3: *n* = 18/32, 56%), HER2‐enriched (*n* = 11/32, 34%) or of luminal‐B‐(like)‐subtype (*n* = 10/32, 31%, Table 2).

**TABLE 2:** Invasive BC characteristics at first and second diagnosis in women with LFS. Data on singular DCIS *n* (first diagnosis) = 3; *n* (second diagnosis) = 4 and malignant phyllodes tumour *n* (first diagnosis) = 1 are not shown. At first diagnosis, one woman had simultaneous bilateral invasive breast cancer while 30 had unilateral BC leading to 32 invasive BC lesions in 31 affected women. At second diagnosis, one women had isolated invasive axillary recurrence and 15 women had unilateral invasive BC ± DCIS

Breast cancer characteristics	First diagnosis *n* (invasive breast cancer lesions) = 32 in 31 affected women	Second diagnosis *n* (invasive breast cancer lesions incl. one isolated invasive axillary recurrence) = 16 in 16 affected women
	*n* (%)	*n* (%)
Initial BC tumour size^a^		
≦2 cm	12 (38)	12 (75)
>2–5 cm	18 (56)	3 (19)
>5 cm	1 (3)	0 (0)
Size after NACT^b^	1 (3)	0 (0)
Axillary recurrence alone	—	1 (6)
Axillary nodal status		
Negative	19 (59)	12 (75)
Positive	13 (41)	3 (19)
Axillary recurrence alone	—	1 (6)
Pathological tumour stage^c^		
0°	6 (19)	2 (12.5)
I	6 (19)	8 (50)
II	17 (53)	3 (19)
III	3 (9)	0 (0)
IV	0 (0)	(0)
Axillary recurrence alone	—	1 (6)
Only clinical stage indicated	0 (0)	2 (12.5)
Molecular subtypes by IHC		
Luminal‐A	4 (13)	3 (19)
Luminal‐B	10 (31)	4 (25)
Triple‐negative	5 (16)	1 (6)
HER2‐positive	11 (34)	8 (50)
+ HR positive	10 (31)	5 (31)
− HR negative	1(3)	2 (13)
HR unknown	0 (0)	1 (6)
Unknown ✥	2 (6)	0 (0)
Grading		
1	1 (3)	1 (6)
2	13 (41)	8 (50)
3	18 (56)	5 (31)
N.I.	0 (0)	2 (13)

Note

*n*, number; N.I., not indicated; HR, hormone receptor; °comprises 2 DCIS (ypTis, ypN0) and six pathological complete remissions (ypT0, ypN0, pCR); IHC—immunohistochemistry; ✥ unknown—HR positive or negative BC without specification of HER2 status; Luminal‐B‐(like)‐subtype was defined as hormone receptor positive, HER2 negative BC with high levels of Ki‐67.

^a^ In case of neoadjuvant systemic therapy (NACT) the initial clinical stage was evaluated/ in case of a primary operation only pathological stages were considered.

^b^ No initial clinical stage indicated, only the pathological stage after NACT was indicated in clinical reports.

^c^ According to the AJCC Anatomic Stage Group (35).

### 3.2 Primary therapy for breast lesions at first diagnosis

Among all 35 women with first diagnosed breast lesions, different kinds of surgery were applied based on tumour board recommendations and patients’ decisions: Breast‐conserving surgery (BCS: *n* = 17/35, 48%) was a common surgical approach. Another nine women received unilateral (*n* = 8/35, 23%) or primary bilateral (*n* = 1/35, 3%) therapeutic ME. Secondary ME due to persistent positive surgical margins were performed in two (*n* = 2/35, 6%) women. In addition, seven (*n* = 7/35, 20%) women were offered unilateral therapeutic ME with simultaneous CPM. This leads to a total number of 18 women receiving any kind of ME (either therapeutically or contralateral prophylactically) as the most common surgical procedure at first diagnosis. MEs were performed as skin‐sparing (*n* = 8/18, 44%) or radically modified ME (*n* = 7/18, 39%) followed by nipple‐sparing ME (*n* = 2/18, 11%) and in one case (*n* = 1/18, 6%) no further specifications on surgical techniques or breast reconstruction were given. 11 women decided towards breast reconstruction: five women each chose primary breast implants (*n* = 5/11, 29%) or expander‐implant reconstruction (*n* = 5/11, 29%), while one (*n* = 1/11, 6%) opted for a tissue flap procedure. Six women chose no breast reconstruction after ME.

Twenty women (*n* = 20/35, 57%) received adjuvant radiotherapy of the breast and/or regional lymph nodes. Focusing on BCS at first diagnosis, radiation was applied in 14 (82%) out of 17 women with BCS, whereas two women (*n* = 2/17, 12%) did not receive adjuvant radiotherapy due to the LFS diagnosis. In one woman with BCS (*n* = 1/17, 6%) no information on adjuvant radiotherapy was available.

The LFS diagnosis was known prior to breast surgery in 2/17 women treated with BCS and in 9/18 women with any kind of ME while all other women received their genetic testing result of a P/LP *TP53* variant after surgery. Among these 9 women with any kind of ME and who were informed about the LFS diagnosis prior to breast surgery, 1 woman chose bilateral therapeutic ME due to bilateral BC, 6 had unilateral ME only and 2 had a unilateral therapeutic ME in combination with CPM.

Sentinel lymph node dissection (SLNB) was performed in 17 out of 35 women (*n* = 17/35, 48%) while axillary lymph node dissection (ALND) was conducted in 13 women (*n* = 13/35, 37%) and secondary ALND in one (*n* = 1/35, 3%). The participant with simultaneous bilateral invasive BC had SLNB on one side and ALND on the other side (*n* = 1/35, 3%) Two women with DCIS (*n* = 2, 6%) and one with a phyllodes tumour (*n* = 1, 3%) did not have any axillary procedure.

Most women with invasive BC (*n* = 28/31, 90%) received chemotherapy ± anti‐HER2 antibody therapy with neoadjuvant (*n* = 13, 46%) or adjuvant regimes (*n* = 15, 54%).

### 3.3 Ipsilateral locoregional recurrence and contralateral BC—second diagnosis

Nineteen women (*n* = 19/35, 54%) had 20 second breast or axillary lesions i.e. locoregional recurrence or a contralateral diagnosis.

In detail, seven women (*n* = 7/35, 20%) had ipsilateral recurrence of invasive BC (in‐breast: *n* = 6/35, 17%, ipsilateral axillary recurrence: *n* = 1/35, 3%), and one (*n* = 1/35, 3%) had ipsilateral (re)‐occurrence of DCIS alone and simultaneous contralateral invasive BC.

In 11 women (*n* = 11/35, 31%) contralateral metachronous BC or DCIS (only DCIS: *n* = 3/35, 27%; invasive BC ± DCIS: *n* = 8/35, 73%) occurred. Pathological and clinical characteristics of second invasive BCs (*n*= 16) are shown in Table 2 compared to the initial BC diagnosis.

Mean age at diagnosis of the second breast lesion was 36 years old (range 24–57 years). Median time between first and second diagnosis was 57 months (95% confidence interval CI: 0.0–114.5). Even though most women with ipsilateral in‐breast recurrence had a previous BCS in opposite to those with prior unilateral therapeutic ME our findings were not statistically significant (*n* = 5/17, 29% vs. *n* = 1/10, 10%; *p* = 0.36, two‐sided Fisher's exact test; one woman with ipsilateral axillary recurrence was excluded).

For surgical treatment of a second BC diagnosis, three out of these 19 women (16%) opted again for BCS (all of them had a metachronous contralateral BC) while thirteen (*n* = 13/19, 68%) received some type of ME. One woman (*n* = 1/19, 5%) with former therapeutic and nipple‐sparing CPM had a nipple‐areola complex excision due to contralateral BC in this region. Two patients (11%) did not undergo a re‐surgical treatment at the time of data analysis due to an ongoing systemic treatment with chemotherapy ± HER2‐targeted therapy. SLNB or ALND was performed in 9 (47%) and 2 (11%) cases.

**FIGURE 2:**
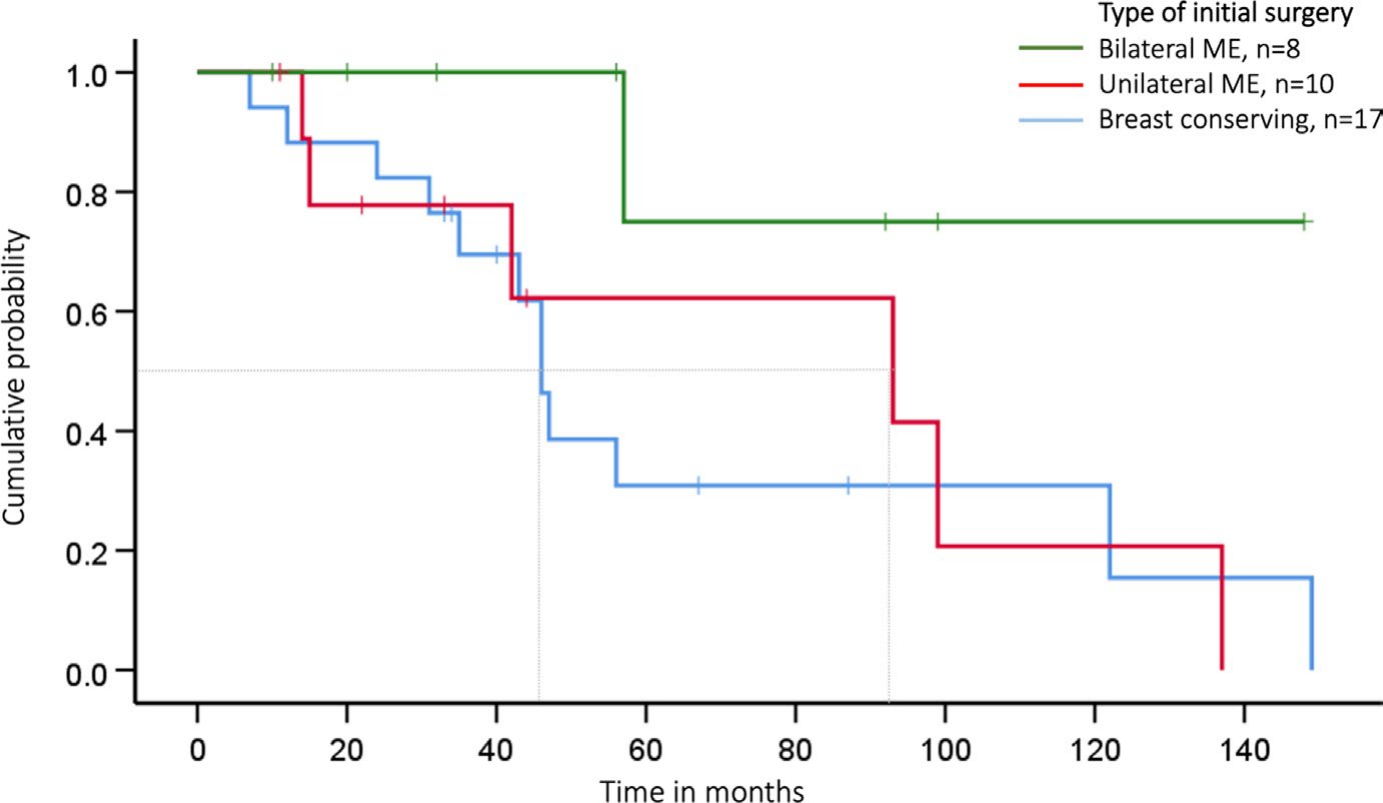
Ipsi‐ and contralateral disease‐free survival depending on type of surgery in women with LFS; we analysed the time between first diagnosis up to one of the events such as ipsilateral BC and/or DCIS, contralateral BC and/or DCIS, both or end of observation time; ME—mastectomy; the grey line marks the median time to recurrence on the *x*‐axis: BCS: 46 vs. unilateral therapeutic ME: 93 months vs. bilateral ME: >140 months; log‐rank test *p* = 0.10

**FIGURE 3:**
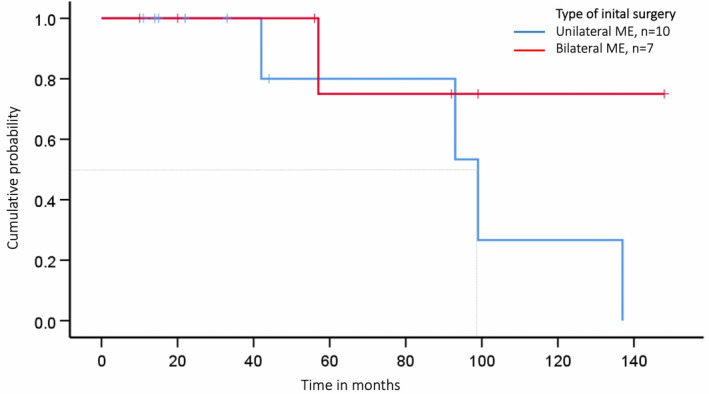
Contralateral disease‐free survival depending on type of mastectomy (ME): For patients with unilateral therapeutic ME alone and unilateral therapeutic ME in combination with simultaneous contralateral ME (bilateral ME) we analysed the time from diagnosis of first BC and/or DCIS to contralateral BC and/or DCIS—one woman with primarily bilateral therapeutic ME was excluded for these subanalyses; ipsilateral events were taken as censored observations. The grey line marks the median time to recurrence on the *x*‐axis: unilateral therapeutic ME: 93 months vs. unilateral therapeutic ME + CPM >140 months; log‐rank test *p* = 0.19

The operation technique influenced the time between first BC diagnosis to either ipsilateral BC and/or DCIS and/or contralateral BC and/or DCIS (Figure 2; Table 3). Patients opting for bilateral ME had a 70% probability of being recurrence‐free over 140 months while those considering breast‐conserving surgery or unilateral ME had only a chance of approximately 20% of being recurrence‐free shortly after 120 months (Figure 2). Contralateral disease‐free survival with respect to the type of ME (unilateral therapeutic ME vs. unilateral therapeutic ME + simultaneous CPM) is presented in Figure 3 and Table 3.

**TABLE 3:** Overview of surgical strategies and (re)‐occurrence of breast tumours in women with LFS. In addition to Figure 2, the time between first diagnosis up to one of the events ipsilateral BC ±/or DCIS, contralateral BC ±/or DCIS, both or end of observation time is represented for each type of breast surgery separately. In addition to Figure 3, contralateral occurrence of BC ±/or DCIS depending on type of mastectomy (unilateral therapeutic ME vs. unilateral therapeutic ME + contralateral prophylactic ME) was analysed. One woman with primary bilateral therapeutic ME was excluded for these subanalyses; ipsilateral events were taken as censored observations

Operational technique	Number of:					No events‐end of observation	Mean time to recurrence in months	95% CI/25% CI	Median time to recurrence in months	95% CI/25% CI
	Patients at first diagnosis	Events at second diagnosis	Ipsilateral recurrence	Contralateral disease	Bilateral disease					
Figure 2:										
Breast‐conserving surgery	17	12	5	6	1	5	67.04	40,55/93,52	46,0	41,49/50,51
Unilateral therapeutic ME	10	6	2^b^	4	0	4	77.99	43,00/112,98	93,0	0,00/192,64
Bilateral ME	8^a^	1	0	1	0	7	125.25	86,63/163,87	NC	NC
Total	35	19^c^	7	11	1	16	79.33	59,37/99,29	57,00	0,00/114,51
Figure 3:										
Unilateral therapeutic ME	10	6	2	4	0	4	77.99	43,00/112,98	93,0	0,00/192,64
Bilateral ME (unilateral therapeutic + contralateral prophylactic)	7	1❖	0	1	0	6	125.25	86,63/163,87	NC	NC
Total	17	7	2	5	0	10	107.11	81,11/133,11	21,69	56,55/141,45

Note

NC—cannot be calculated ❖ Contralateral BC occurred in the nipple‐areolar‐complex in one patient with formerly therapeutic nipple‐sparing ME and simultaneously contralateral prophylactic nipple‐sparing ME.

Abbreviations: CI, confidence interval; ME, mastectomy.

^a^ Including one woman with bilateral therapeutic mastectomy and seven women with unilateral therapeutic mastectomy in combination with contralateral prophylactic mastectomy.

^b^ One woman with in‐breast recurrence after nipple‐sparing mastectomy and one woman with axillary recurrence after radical mastectomy. ^c^One woman with primary bilateral therapeutic M was excluded for these subanalyses

^c^ One woman with primary bilateral therapeutic M was excluded for these subanalyses

### 3.4 Other preventive surgery and satisfaction with surgical results

**FIGURE 4:**
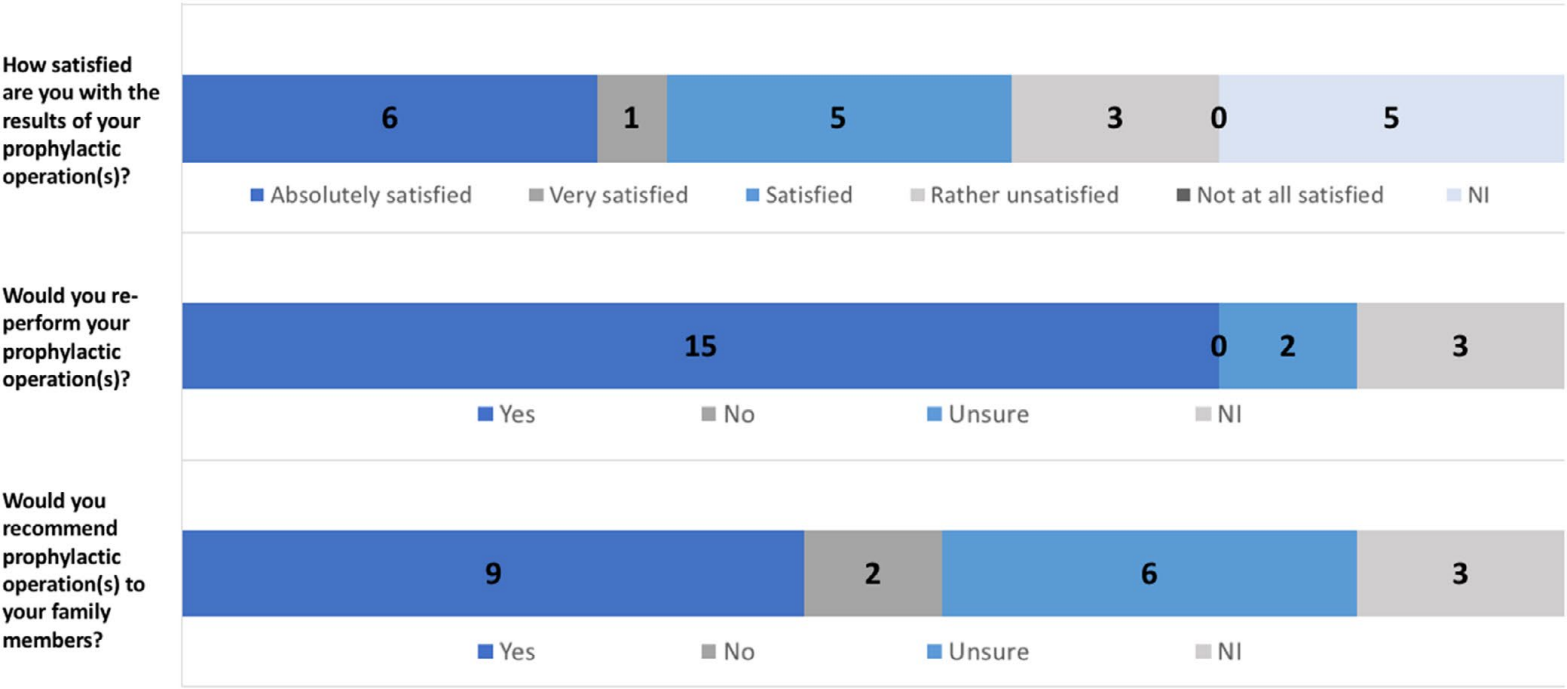
Satisfaction with prophylactic operation results; N.I., not indicated

One woman with LFS opted for BPM in combination with a PBSO while 19 women with primary BC or a second BC diagnosis underwent any kind of PO (*n*(any kind of PM) = 16, *n*(any kind of PM + PBSO) = 3). The satisfaction with the surgical results is presented in Figure 4. We could see a tendency that women choosing PO were slightly older than women without PO (39.7 ± 10.2 vs. 37.8 ± 12.4 years; *t*‐test *p* = 0.59).

## 4 DISCUSSION

This analysis is, to our knowledge, the first to address surgical BC treatment and risk‐reducing surgery among women with LFS. In accordance with previous studies,^25^, ^28^, ^29^, ^31^, ^32^ we observed more aggressive BC subtypes with high nuclear grade and positive HER2‐status or luminal‐B‐(like)‐phenotype in women with LFS, necessitating neo‐ or adjuvant‐systemic therapy. At first diagnosis, 53% and 9% of affected women had pathological tumour stages II or III, often nodal positive (41%). In case of recurrence, most women presented with lower tumour stages which might be due to intensified follow‐up including breast MRI. The internationally recommended LFS‐specific cancer surveillance programmes were not applied prior to BC diagnoses for most subjects as in most cases the LFS diagnosis was unknown prior to the first BC diagnosis. In general, genetic analyses were performed as part of an HBOC multigene panel analysis including *TP53*.

In our cohort, only 11 women (31%) with any kind of a breast lesion knew about their P/LP *TP53 g*ermline variant prior to their first oncological treatment. It has been previously emphasised that knowledge of a detailed family history preoperatively and rapid first priority genetic testing in “fast track mode” even before surgery can optimise care especially if LFS is suspected e.g. in women ≤30 years old and especially in HER2 enriched BC in young women and should be discussed prior to therapy.^11^, ^30^, ^32^ On the other hand, overburdening women with decision‐making about secondary prophylactic measures in the temporal context of primary therapy must be considered and requires further investigation.

In general, the LFS diagnosis influences BC treatment. Our data showed that the LFS diagnosis was rarely known prior to BCS. Normally, ME is discussed in order to avoid breast radiation after BCS due to the risk of radiation‐induced secondary malignancies, such as sarcoma, small lung cancer or thyroid cancer, which is described in up to 33% of individuals with LFS.^22^, ^23^, ^25^, ^26^, ^27^ Furthermore, the risk of in‐breast recurrence after radiation has been addressed previously by Heymann et al., who found an ipsilateral “in‐field relapse” of BC in three out of six patients after a median follow‐up of 6 years.^23^ A recent study by Le et al. with a follow‐up of 12.5 years described a lower risk for locoregional BC recurrence in the chest wall after post‐ME radiotherapy (*n* = 1/8, 13%) and for secondary malignancies (sarcomas: *n* = 1/18, 6%; thyroid cancer: *n* = 1/18, 6%) and pointed out that there is no absolute contraindication for radiotherapy.^25^ In our cohort, all 7 women with ipsilateral in‐breast recurrence had former radiotherapy of the breast (5 women with former BCS + radiation and 2 with former ME + radiation, data not shown) thus probably supporting the finding of Heymann et al.. Even in case of a second BC disease, 16% of our affected study participants opted again for BCS. Time after last radiation varied due to individual treatment schedules and was not further studied. Adjuvant radiation is generally recommended to start within 8 weeks after BC surgery according to the German national S3 guideline with no differentiation or further specifications for individuals with LFS.^37^ At most centres, radiation is typically offered 4–8 weeks after surgery. Its precise impact ought to be addressed in further studies. These findings emphasise that individual treatment strategies for LFS‐BC patients are necessary, especially before ≤30 years or in young Her2 positive BC patients. Though most participants had no knowledge on their *TP53* status at first diagnosis the next surgery should take this information into account and adapt individual treatment strategies. Further studies with larger collectives e.g. through registers in order to achieve LFS‐specific surgical, radiation and systemic protocols are needed. Furthermore, evidence for rare cancer predisposition syndromes should be included in S3 BC guidelines.

Due to the risk of contralateral BC in P/LP *TP53* germline variant carriers described in literature,^2^, ^15^, ^17^ CPM might be justifiable in case of primary unilateral BC.^22^ This corresponds to the high rate of contralateral BC in our cohort. In a study using whole body MRI for baseline surveillance in individuals with LFS, Ballinger et al. described that only two out of 264 women were diagnosed with BC. However, almost half of the study population had uni‐ or bilateral ME prior to study participation (*n* = 127/264, 48%) which might explain the low rate of BC or BC recurrence in their cohort.^38^ In our series, the rate of uni‐ or bilateral‐MEs in primary BC was comparable with 51% (*n* = 18/35 ME performed) while we observed a significantly higher rate of locoregional recurrence and contralateral BC. It should be noted that there might have been a selection bias due to the inclusion‐ and genetic testing criteria of the GC‐HBOC or due to the fact that this cohort represents a volunteer cohort. Only one healthy carrier had a prophylactic bilateral ME in combination with a prophylactic PBSO while 19 women with primary or secondary BC underwent any kind of PO in our cohort. Saya et al. described a rate of 33.3% of female *TP53* germline P/LP variant carriers with risk‐reducing operations or ME for previous BC.^39^ Our findings are in accordance with reported rates of CPM in P/LP *BRCA1*/*2* variant carriers ranging between 0% and 49.3% while BC history, an aggregation of familial BC cases and a young age at cancer onset were predictive factors for bilateral risk‐reducing ME or CPM.^40^, ^41^, ^42^, ^43^ Women opting for PO in our cohort were slightly older and most of them had a prior BC diagnosis. As LFS‐BC often occurs below the age of 31, prophylactic surgery might be even justifiable before the age of 30 in healthy women meeting classical LFS criteria. In the case of BC, therapeutic and CPM should be considered as an option especially for young women with BC and LFS after discussion of concurrent risks.

Our data suggest that surgical strategies might influence median time to ipsilateral and contralateral recurrence and thus survival times but further prospective studies are needed. In case of BCS ipsilateral BC re‐occurred earlier compared to women treated with unilateral therapeutic ME. Additionally, women with a therapeutic ME alone suffered from contralateral recurrence earlier than women treated with therapeutic ME in combination with a CPM. Large scale studies in other BC populations have shown that surgical techniques such as nipple‐sparing ME guarantee a high oncological safety with well‐accepted cosmetic results which might justify prophylactic procedures in high‐risk populations as in LFS patients. However, those conclusions for LFS patients require larger studies as our cohort only included two women with nipple‐sparing ME.^44^, ^45^

We could demonstrate that satisfaction with surgical results was high and most study participants would even recommend it to family members presuming a legitimate procedure in terms of psychological and cosmetic aspects. While studies focusing on PO among *TP53* germline variant carriers are missing, systematic reviews in subjects with other high penetrant BC genes such as *BRCA 1 and 2* described high satisfaction with PO, good quality of life and excellent cosmetic results whereupon 90% would repeat the surgical procedure 20 years postoperatively.^40^, ^46^, ^47^ However, if postoperative complications and subsequent surgeries were performed, it had a negative perception of PO.^48^, ^49^, ^50^ Prospective studies are desirable to address the best surgical technique and the respective satisfaction in women with LFS. The best medical and psychosocial outcome e.g. overall survival, quality of life or patient's satisfaction with surgical results and PO decisions shall be achieved.

Contrary to the current international guidelines four of our study participants underwent PBSO. In order to avoid unnecessary interventions, individuals with rare cancer predisposition syndromes should be counselled in specialised centres where a multidisciplinary concept with evidence‐based medicine and a broad expertise with respect to the underlying syndrome is available to avoid overtreatment and to offer maximum supportive care.

This study is limited by its small sample size and its retrospective character. In addition, in most participants, the LFS diagnosis was unknown prior to breast surgery. A prospective study is necessary in order to confirm our results and to investigate further detailed analysis such as nipple‐sparing procedures or time between locoregional recurrence and radiation. Current studies are underway to better reflect the broad phenotypic spectrum associated with L/LP variants of *TP53*.^51^

## 5 CONCLUSION

Aggressive BC subtypes are detected among women with LFS. The surgical treatment and CPM may increase the time to a second BC diagnosis whereas larger studies are needed for further statements. Since LFS is a rare cancer predisposition syndrome, national and international tumour registries are essential to achieve more profound, individualised and evidence‐based statements for treatment options including prophylactic operations.^52^ Individual competing tumour risks and long‐term medical as well as psychosocial outcome need to be taken into consideration in risk and preventive counselling.

## CONFLICTS OF INTEREST

None.

## ETHICAL APPROVAL STATEMENT

Human investigations were performed after approval by an institutional ethics committee and in accordance with the principles outlined in the Declaration of Helsinki. Informed consent was obtained from each study participant.

## Data Availability

The authors declare that the data supporting the findings of this study are available within the article.
